# Impact of Vision and Hearing Impairment on Cognition and Loneliness: Evidence From the Mexican Health and Aging Study

**DOI:** 10.1093/geroni/igab046.653

**Published:** 2021-12-17

**Authors:** Kimberly Hreha, Rafael Samper-Ternent, Brian Downer, Joshua Ehrlich, Paige Downer, Timothy Reistetter, Heather Whitson

**Affiliations:** 1 Duke University, Durham, North Carolina, United States; 2 The University of Texas Medical Branch, Galveston, Texas, United States; 3 University of Texas Medical Branch, Galveston, Texas, United States; 4 University of Michigan, University of Michigan, Michigan, United States; 5 University of Texas Health Science Center at San Antonio, Dickinson, Texas, United States; 6 Duke University School of Medicine, Durham, North Carolina, United States

## Abstract

Poor vision and hearing have been associated with lower cognitive function and greater social isolation (i.e., loneliness) among older adults. However, this evidence is based largely on data from non-Hispanic populations. Therefore, we investigated whether self-reported vision and hearing was associated with cognitive function and loneliness in a nationally-representative study of Mexican adults aged 50 and older in Wave 3 of the Mexican Health and Aging Study. The final sample included 12,426 participants. The majority were female (58%), and the mean age was 67. Self-reported vision and hearing status were categorized as excellent-very good [ref], good, and fair-poor. Measures for global cognition, memory, and non-memory cognition were calculated using z-scores based on nine cognitive tests. Participants who reported frequently feeling a lack of companionship, left out, or isolated were categorized as feeling lonely. All analyses controlled for age, sex, and years of education. Participants with fair-poor vision had lower global (β= -0.06, p <.01), memory (β= -0.07, p <.01), and non-memory cognition (β= -0.06, p <.01) than participants with excellent-very good vision. In addition, participants with fair-poor hearing had higher non-memory cognition (β= 0.03, p <.05) but not global cognition (β=0.02) or memory (β=0.001). Fair-poor vision (OR=1.53, 95% CI=1.25-1.87) but not fair-poor hearing (OR=1.16, 95% CI=0.97-1.38) was associated with higher odds of being lonely. Poor vision may be a potentially modifiable risk factor for lower cognition and loneliness among Mexican adults. Future research should incorporate robust measures of sensory health and investigate the longitudinal association between vision, cognition, and loneliness.

